# Hot-Pressed Wet-Laid Polyethylene Terephthalate Nonwoven as Support for Separation Membranes

**DOI:** 10.3390/polym11101547

**Published:** 2019-09-23

**Authors:** Lei Xia, Quping Zhang, Xupin Zhuang, Shuo Zhang, Chengpu Duan, Xiaoyin Wang, Bowen Cheng

**Affiliations:** 1 State Key Laboratory of Separation Membranes and Membrane Processes, Tianjin Polytechnic University, Tianjin 300387, China; xia1983@163.com (L.X.); zerofone@163.com (Q.Z.); zhxupin@tjpu.edu.cn (X.Z.); 2 School of Textile Science and Engineering, Tianjin Polytechnic University, Tianjin 300387, China; zs982975971@163.com (S.Z.); cp_duan327518@163.com (C.D.); 3 School of Mathematical Science, Tianjin Polytechnic University, Tianjin 300387, China; 4 Tianjin University of Science and Technology, Tianjin 300222, China

**Keywords:** separation membrane, polyethylene terephthalate, wet-laid, hot pressing, response surface methodology

## Abstract

In this work, a polyethylene terephthalate (PET) nonwoven support was prepared by wet-laid and hot-press technology and used as support for separation membranes. The properties of the PET nonwoven support were studied to determine the effect of hot-pressing parameters and PET fiber ratio, and were optimized by response surface methodology. Result showed that the PET nonwoven support with 62% low melting point PET (LPET-180) fibers obtained satisfactory properties and structure after hot pressing at 220 °C under the pressure of 9 MPa for 20 s. The response surface analysis indicated that the temperature and time of hot pressing and the fiber ratio were the most important factors affecting the strength and air permeability of the PET nonwoven support. After hot pressing, the PET nonwoven support exhibited interconnected structure, small pore size, low porosity, and high strength. Then phase inversion technique was applied to prepare a polysulfone (PSF) layer on the PET nonwoven support and an ultra-thin polyamide (PA) active layer was prepared by interfacial polymerization on the PSF layer. The practicality of PET nonwoven support was verified by testing the pure water flux and retention of the PA composite membrane and the structural change of the PA composite membrane before and after use. The results proved the feasibility and remarkable application prospects of hot-pressed wet-laid PET nonwoven support as support for separation membranes.

## 1. Introduction

Water is the backbone of the global economy; a high-quality and sustainable supply is essential for production and livelihood [1]. It is still a challenge for water purification technology to efficiently and effectively produce clean water through environmentally friendly ways [2]. As a new separation method, membrane separation technology exhibits the advantages of low energy consumption, high efficiency, simple operation, easy scale-up, less land occupation, and environmental friendliness. This technology is one of the most important new technologies in the 21st century and has been widely used in water treatment in recent years.

In general, a separation membrane such as a nanofiltration or reverse osmosis membrane is a composite of a dense ultra-thin active layer (AL), a porous polymer support layer (SL) and a fabric support [3,4,5,6]. Commonly, woven or nonwoven fabrics are used as the support to increase the membrane mechanical strength for application [7,8]. The homogeneity of the fabric support is very important to avoid pinhole defects in the formation of the SL [9,10,11,12,13,14,15,16,17]. In addition, a fabric support with pores that are too large can easily cause the penetration of the polymer solution through the fabric support and is unable to hold, resulting in the inability of the fabric support to hold the polymer solution to form a SL [18]. Simultaneously, the use of a fabric support with pores that are too small results in the poor adhesion of the SL and thus easily causes delamination of the SL [18]. As a result, the pore size of the fabric support should be carefully tuned. To date, the most studies have focused on the preparation and performance optimization of the SL and AL, whereas limited research on the fabric support has been reported.

The wet-laying technique is widely used for fabricating nonwoven [19]. In this process, a large amount of water is used as a medium to obtain a uniform fiber suspension and fiber web after dehydration. Wet-laid nonwoven fabric has several advantages: the inner fibers of the formed web under the condition of water flow are distributed in three dimensions with good uniformity; it has a good disorderly arrangement of fibers; and has large pore structure. However, the fiber web is generally loose because of the low bonding strength between fibers. Therefore, wet-laid nonwoven supports are physically or chemically treated or processed to overcome these shortcomings and improve the mechanical properties.

Hot pressing, which typically uses one or two pairs of heated steel rollers or other molds to heat and pressurize the fiber web, is a suitable approach for strengthening wet-laid nonwoven fabrics. It allows the melting, flowing, and diffusion of low-melting-point fibers in the web. Upon cooling, the fibers are bonded, and the web is strengthened to form hot-pressed nonwoven supports. In this process, melted fibers not only act as a binder to bond and protect other fibers, but also decrease the pore size of the nonwoven fabric. This technique is considered to be a promising approach to fabricating fabric supports because of its fast production speed, lack of waste water, waste gas, and solid waste, and suitability for reinforcement of thin nonwoven fabric. The bonding strength of wet-laid nonwoven can be enhanced through hot pressing to improve their mechanical properties. Moreover, this method can reduce the pore size of wet-laid nonwoven fabrics and decrease their porosity.

In this study, polyethylene terephthalate (PET) fibers were used to prepare supports for a separation membrane through a wet-laying process and hot-pressing technology. PET fibers are commercialized and well-known semi-crystalline aromatic polyesters for several applications worldwide [20,21,22,23,24]. The success of PET fibers can be attributed to their excellent balance between mechanical, chemical, and thermal properties and production cost. These fibers can also be employed to easily obtain different melting points by copolymerization of the second monomer [25,26,27]. PET fibers with low melting point (LPET) were blended to bond the fibers after hot pressing.

Herein, we prepared PET nonwoven support (PNS) using wet-laying and hot-pressing techniques, investigated the effect of different hot-pressing parameters on the properties of the PNS, and finally established a method to optimize its mechanical properties and air permeability. The PNS before and after hot pressing was analyzed and compared to explore the variations caused by hot pressing. To further demonstrate the applicability of PNS, a separation membrane was prepared by assembling the PNS with a polysulfone (PSF) layer and an ultra-thin polyamide (PA) active layer. The flux and retention of the composite membrane are discussed, and the structures of composite membrane before and after use were also investigated.

## 2. Material and Experiment

### 2.1. Materials

Conventional PET fiber (PET-260, 0.69dtex, 6 mm long, melting point of 260 °C) and low-melting-point PET fiber (LPET-180, 1.84dtex, 6 mm long, melting point of 180 °C) were obtained from Baohong New Material Limited (Guangzhou, China). A commercial fabric support was obtained from Freudenberg BaoLing Nonwoven Co., Ltd. (Suzhou, China). Polysulfone (PSF; P-3500 LCD MB7, 77–83 kDa) was purchased from Furun Plastic Technology Co., Ltd. (Shanghai, China). NaCl, MgSO_4_, *N,N*-dimethylformamide (DMF, Analytical purity), *m*-phenylenediamine (MPD, Analytical purity), camphor sulfonic acid (Analytical purity), triethylamine (TEA, Analytical purity), trimesoyl chloride (TMC, 98%), and polyethylene oxide (PEO, 5000 kDa) were acquired from Sigma-Aldrich (Shanghai, China). *N*-Heptane (Analytical purity) was obtained from Jiangtian Chemical Co., Ltd. (Tianjin, China). All chemicals were used as received without further purification.

### 2.2. Preparation of PET Nonwoven Support

**Figure 1:**
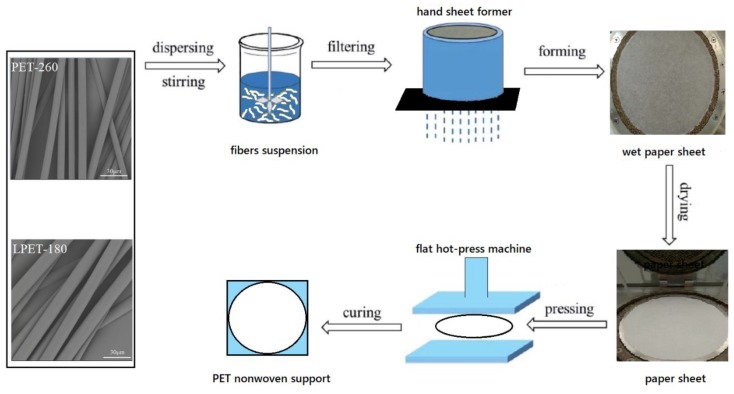
Schematic of the polyethylene terephthalate (PET) nonwoven support.

PET nonwoven support was fabricated through wet-laying, followed by hot pressing. Firstly, LPET-180 fibers and PET-260 fibers were mixed under stirring with 0.5% PEO as dispersant to form a fiber suspension. The suspension was poured into the container of a hand sheet former to drain thoroughly and form a wet nonwoven support sheet. The sheet was dried at 110 °C and hot pressed with a flat hot-presser to obtain a PET nonwoven support. The schematic of preparation is shown in Figure 1. The fiber ratio and hot-pressing parameters were examined to adjust the structure of the PNS.

### 2.3. Fabrication of PSF Ultrafiltration Membrane and PA Composite Membrane

A PSF ultrafiltration (UF) membrane was prepared on the PNS by wet phase separation. For this, 22 wt.% of PSF was dissolved in DMF at 25 °C and the polymer solution then stood at room temperature for 8 h. Subsequently, the PNS was attached to a clean glass plate using adhesive tape, and the PSF solution was casted on the PNS using a casting knife with a gate height of 150 μm. The whole composite was immediately immersed a coagulation bath filled with DI water. The polymer support was washed and kept in DI water at room temperature before use.

The PA active layer was prepared on top of the PSF UF membranes via interfacial polymerization. The PSF UF membrane was immersed in a 2.0 wt.% MPD solution with 2.6 wt.% camphor sulfonic acid and 1.1 wt.% TEA for 2 min. Then, the MPD-saturated support membrane was immersed in *N*-heptane solution with 0.1 wt.% TMC for 1 min. Finally, the fabricated PA composite membranes were rinsed thoroughly and stored in DI water prior to performance testing.

### 2.4. Characterizations and Analysis

#### 2.4.1. Characterization of the PET Nonwoven Support before and after Hot Pressing

The morphology of the PET nonwoven support was evaluated using a scanning electron microscope (SEM, FlexSEM1000, Hitachi, Japan).

Fourier-transform infrared spectroscopy (FTIR) measurements were performed on a Nicolet iS50 FTIR spectrometer (Thermo Fisher Scientific, Waltham, MA, USA) to determine chemical changes before and after hot pressing. All samples were ground, mixed with potassium bromide, and pressed to form pellets. The FTIR spectra were recorded in the range of 4000–500 cm^−1^.

X-ray diffraction (XRD) measurement was carried out on a D8 Discover with GADDS (BRUKER AXS Co., Ltd., Karlsruhe, Germany) to observe the crystallinity of the PET nonwoven support before and after hot pressing.

#### 2.4.2. Mechanical Characterization

The tensile index (TI) and breaking elongation (BE) of PET nonwoven support were obtained by a YG028 Universal Material Testing Machine (Wenzhou Fangyuan Instrument Co., Ltd., China). The PNS had a size of 100 mm × 50 mm, and the test was performed at a strain rate of 10 mm/min. All the measurements were conducted three times at room temperature. TI and BE were calculated as follows:

TI (N·m·g^−1^) = 1000 × *F*/(*L_w_* × *g*), (1)

BE = (*L_b_*/*L*_0_ − 1) × 100%, (2)

where *F* (N/5cm) is the average tension resistance; *L_w_* (mm) and g (g/m^2^) are the width and weight of the sample, respectively; and *L*_0_ and *L_b_* are the initial length and fracture length of the sample, respectively.

The tightness of the PNS was assessed by cutting the nonwoven support into 30 mm × 30 mm pieces. The sample was weighed, and its thickness was measured by a Digital Thickness Tester (Wenzhou Fangyuan Instrument Co., Ltd., China). The tightness of the PET nonwoven support was calculated as follows:

Tightness (g·m^−3^) = *M*/(*S* × *H*), (3)

where *M* (g), *S* (m^2^), and *H* (m) are the weight, area, and thickness of the PET nonwoven support, respectively.

Air permeability (AP) was adopted to characterize the pore size change and reduce the test time. A YG461H Automatic Fabric Pressure Permeability Instrument (Ningbo Textile Instrument Factory, China) was used to measure the AP.

#### 2.4.3. Response Surface Analysis

Response surface methodology (RSM) was used to study the hot-pressing process of PNS and optimize the hot-pressing parameters by Software Design-Expert Version 11 (Stat-Ease, Minneapolis, MN, USA). The pore structural parameters of the optimized PET nonwoven support were measured by a mercury porosimeter (Auto pore IV9500, Micromeritics Instrument Co., Ltd., Norcross, GA, USA).

#### 2.4.4. Characterization of PA Composite Membrane

The morphology of PA composite membrane before and after use was determined using an AFM (NT-MDT Prima, Bruker, Germany).

The flux and retention of PA composite membrane were measured on an assembled dead-end stirred cell system. The effective area of the membrane was 19.635 cm^3^. During the measurement, a variable-speed diaphragm pump (DP-130, Xinxishan Industrial Co., Ltd., Wenzhou, China) was used to control the transmembrane pressure. The membrane was tested at 0.2 MPa. The flux is calculated as follows:

*J* (L·m^−2^·h^−1^) = *V*/(*A* × *T*), (4)

where *V* (L) is the volume of the permeated water, *A* (m^2^) is the active area of the membrane, and *T* (h) is the penetration time.

A 2000 ppm MgSO_4_ solution and 2000 ppm NaCl were used as aqueous feed solutions. The conductivity of the permeates was measured using a digital conductivity meter (DDSJ-308A, Inesa Limited Co., Ltd., Shanghai, China). Retention (*R*) was calculated as follows:

*R* = (1 − *C_p_*/*C_f_*) × 100%, (5)

where *C_f_* and *C_p_* are respectively the conductivity of the feed and permeate.

## 3. Results and Discussion

### 3.1. Morphology and Interface Combination of the PET Nonwoven Support

The PET nonwoven support was prepared using high-melting-point PET fibers and low-melting-point PET fibers by wet-laying and hot pressing. The support had a complex network interleaving structure, which directly affected its physical properties. The comprehensive properties of the support not only depended on the properties of PET-260 fibers and PET-180 fibers, but also on the interface combination between them. The interface as a link between two kinds of fibers is an important microstructure of the nonwoven support.

**Figure 2:**
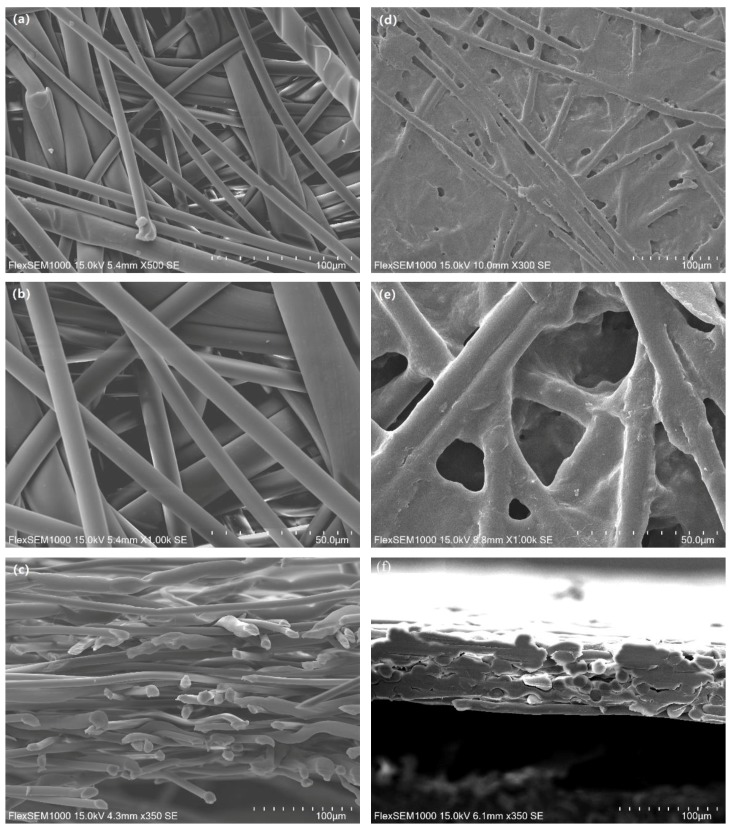
SEM images of the surface of the PET nonwoven support (**a**,**b**) before and (**d**,**e**) after hot pressing, and cross section (**c**) before and (**f**) after hot pressing.

Figure 2 shows the SEM images obtained to observe the surface and cross section of PET nonwoven support before and after hot pressing. Before hot pressing (Figure 2a–c), PET-260 and LPET-180 fibers intertwined with each other, and their arrangement was loose and disordered. The overall structure of PET nonwoven support was porous and loose. The structure of the nonwoven support became dense after hot pressing (Figure 2d–f). In this process, LPET-180 fibers were melted and bonded the fiber network together. PET-260 fibers, as reinforcement, deformed partially at high temperatures and pressure levels, resulting in increased contact area between fibers. The increased area is conducive to the formation of bonding force between fibers. PET-260 fibers were embedded in the deformed LPET-180 fibers to maintain the relative position between the fibers. The formed structure of PET nonwoven support was similar to a reinforced concrete structure, thereby indicating the mechanical properties of the nonwoven support [28].

**Figure 3:**
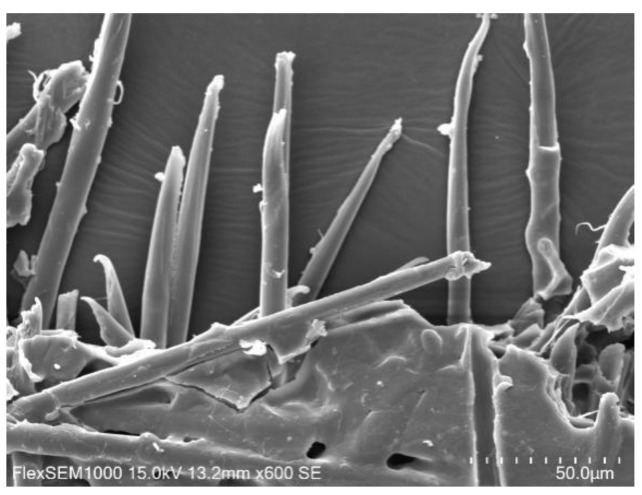
SEM image of tensile fracture morphology of the PET nonwoven support.

The PET-260 fibers were pulled out completely at the tensile fracture position of the nonwoven support (Figure 3). Some PET-260 fibers underwent slight physical deformation at high temperatures and pressure levels, but their surfaces were relatively clean and had no PET-180 fibers. This is mainly attributed to the following: PET-260 fibers are highly crystalline polymers, the fibers have a molecular chain surface that contains only hydrogen bonds, and there is a steric effect of the benzene ring groups. As such, the PET-260 fibers had high chemical inertia on the surface and had difficulty in chemically bonding with PET-180 fibers.

**Figure 4:**
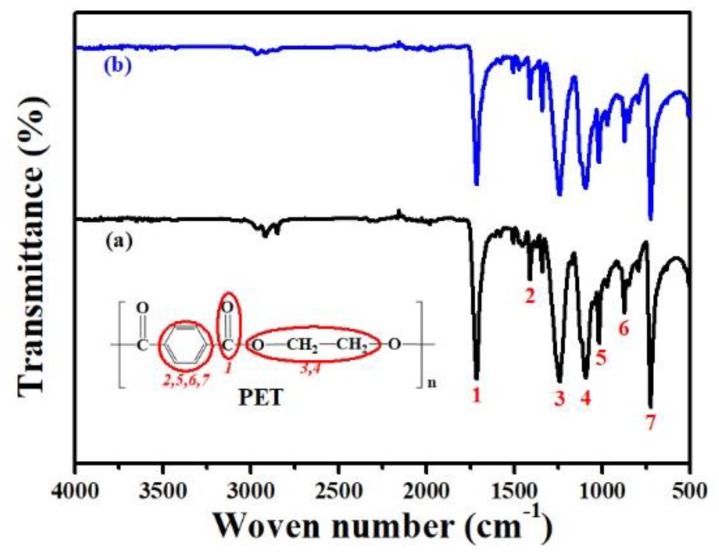
FTIR spectra of PET nonwoven support: (**a**) before and (**b**) after hot pressing.

To further characterize the physical bonding between PET-260 short fibers and low-melting-point LPET-180 fibers in the PET nonwoven support, the FTIR spectrogram of the PET nonwoven support is presented in Figure 4. Seven major peaks were observed and attributed as follows: (1) 1710 cm^−1^, the stretching of –C–O present in the ester group; (2) 1409 cm^−1^, the characteristic band of the disubstituted benzene ring; (3) 1243 cm^−1^ and (4) 1092 cm^−1^, the asymmetric stretching of –C–C–O and O–C–C, respectively; (5) 1012 cm^−1^, (6) 866 cm^−1^, and (7) 720 cm^−1^, the presence of the benzene ring, the aromatic para-substitution, and the C–H vibrations from the aromatic structures, respectively. Similar results are found in literature for PET membranes fabricated by other techniques [29,30,31,32]. The peak shape of infrared spectra before and after hot pressing was similar, and the peak positions of groups resembled one another. This finding indicates that the main chemical groups of the support fabric before and after hot pressing were uniform, and the molecular structure of the nonwoven support did not change significantly during hot pressing.

### 3.2. Effect of Process Parameters on PET Nonwoven Support

The PET nonwoven support prepared by wet-laying had uniform fiber distribution and large pores. However, due to the characteristics of PET fibers, mechanical strength could not be increased by relying on their own hydrogen bonding and mechanical entanglement as in natural pulp. Hot pressing can improve the bonding fastness between PET fibers and effectively increase the tightness of PET nonwoven support, thereby enhancing its overall mechanical strength and reducing its pore size. To further understand the influence of the hot-pressing process on the PET nonwoven support, the effects of hot-pressing parameters such as temperature, pressure, time and fiber ratio on the mechanical strength and pore size of the nonwoven support are further discussed in the next section.

#### 3.2.1. Fiber Ratio

**Figure 5:**
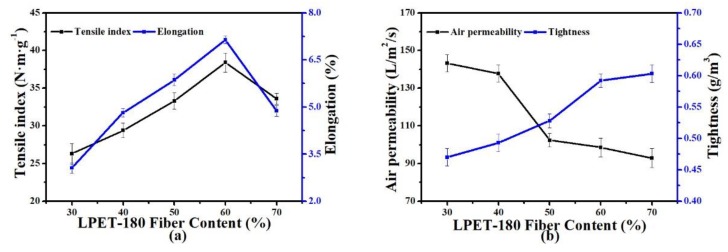
Effect of fiber ratio on the properties of the PET nonwoven support (hot-pressing temperature: 200 °C; hot-pressing pressure: 6 MPa; hot-pressing time: 10 s): (**a**) Effect of fiber ratio on tensile index and elongation, and (**b**) effect of fiber ratio on air permeability and tightness.

The physical strength of nonwoven support depends on fiber strength and the bond strength among fibers. In the PET nonwoven support, PET-260 fibers were the skeleton that provided the strength to the entire nonwoven support, whereas as the bonding fiber LPET-180 determined the bond strength among fibers. The performance of the nonwoven support can be greatly improved by completely utilizing their characteristics. Figure 5 shows the effect of fiber ratio on the properties of the nonwoven support. The AP decreased gradually, but the TI and elongation of the nonwoven support initially increased and then decreased with increased content of added LPET-180. At 30% LPET-180 fiber content, the bond strength among fibers was the main factor affecting the strength of the nonwoven support, but the bond strength was insufficient at this time. With increased LPET-180 fiber content, the TI and elongation rapidly rose to 40–60% and achieved the maximum value at 60% LPET-180 content. The PET-260 fiber’s strength gradually became the main factor influencing the mechanical strength. To a certain extent, the change of AP reflected the change in pore size of the nonwoven support (i.e., a lower AP meant a smaller pore size). We observed that pore size decreased sharply with increased content of LPET-180 from 30% to 50% because the PET nonwoven support with a higher content of LPET-180 was more easily compacted during hot pressing resulting in greater tightness and lower pore size. However, when the LPET-180 content was 70% of the total fiber content, PET-260 fiber, comprising only 30% of the entire fiber content, was insufficient to provide sufficient fiber strength. Consequently, the TI and elongation and pore size of the PET nonwoven support decreased due to the large amount of melted LPET-180.

#### 3.2.2. Hot-Pressing Temperature

**Figure 6:**
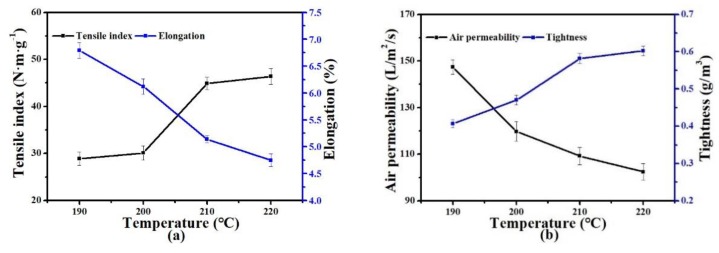
Effect of hot-pressing temperature on the properties of PET nonwoven support (hot-pressing pressure: 6 MPa; hot-pressing time: 10 s; and PET-260/LPET-180: 7/3 (*w/w*)): (**a**) effect of temperature on tensile index and elongation, and (**b**) effect of temperature on air permeability and tightness. LPET-180: low-melting-point PET fiber (i.e., melting point 180 °C); PET-260: PET fiber with melting point of 260 °C.

The effects of temperature on the mechanical properties of the PET nonwoven support are shown in Figure 6. With increased hot-pressing temperature, the mechanical properties of the nonwoven support increased, and the best TI was obtained at 220 °C, whereas AP gradually decreased and thus pore size decreased. During the increase in hot-pressing temperature, the melted LPET-180 with better fluidity bonded well with PET-260 fibers by inducing plastic deformation to a certain extent. The entire structure of the nonwoven support became dense, and the hot-pressing temperature markedly increased the TI (Figure 6a). However, preparing the PET nonwoven support at >220 °C was unsuitable because the nonwoven support became brittle as a result of the decrease in LPET-180 fibers’ elasticity and overconcentrated local stress at >220 °C.

#### 3.2.3. Hot-Pressing Pressure

**Figure 7:**
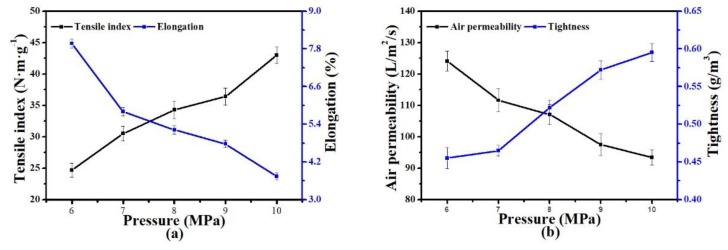
Effect of hot-pressing pressure on the properties of PET nonwoven support (hot-pressing temperature: 200 °C; hot-pressing time: 20 s; PET-260/LPET-180: 7/3 (*w/w*)): (**a**) effect of pressure on tensile index and elongation, and (**b**) effect of pressure on air permeability and tightness.

Figure 7 shows the influence of hot-pressing pressure on the mechanical properties of the nonwoven support. With the increase of hot-pressing pressure, the TI of the nonwoven support gradually increased, and AP gradually decreased. However, the change rates were not particularly drastic compared with those of elongation and tightness. The reasons were as follows: On the one hand, the raise in pressure significantly affected the compaction of melted fibers, which greatly improved the tightness of nonwoven support and reduced the pore size. On the other hand, the bonding strength among fibers improved, compared with low-pressure conditions, more PET-260 fibers in nonwoven support prepared under high-pressure conditions broke rather than slipped at the tensile fracture point, resulting in decreased elongation and enhanced mechanical properties.

#### 3.2.4. Hot-Pressing Time

**Figure 8:**
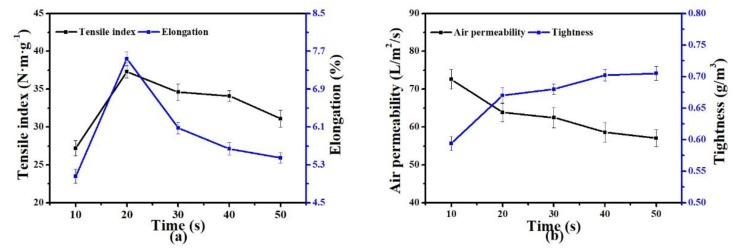
Effect of hot-pressing pressure on the properties of PET nonwoven support (hot pressing temperature: 190 °C; hot pressing pressure: 8 MPa; PET-260/LPET-180: 3/7 (*w/w*)): (**a**) effect of time on tensile index and elongation, (**b**) effect of pressure on air permeability and tightness.

Figure 8 shows the effect of hot-pressing time on the performance of PET nonwoven support. AP slowly decreased but the TI of the nonwoven support initially increased rapidly and then descended gradually with increased hot-pressing time. The short hot-pressing time was insufficient to induce complete melting and flowing of LPET-180 fibers. Moreover, the TI and elongation of the PET nonwoven support obtained by hot pressing for 10 s was very low. Conversely, when the hot-pressing time exceeded 20 s, the strength of PET-260 fibers was lost under the combined action of high temperature and pressure for a long time, resulting in decreased overall strength and elongation of the nonwoven support decline. However, with prolonged hot-pressing time, the low-melting-point fibers sufficiently melted and flowed, causing the structure of nonwoven support to become closer and have decreased pore size and AP.

### 3.3. Regression Equation Prediction Model of TI and AP

To further study the hot-pressing process of the PET nonwoven support, response surface methodology was used to study this process. Based on the results of our single-factor experiments, the parameters of hot-pressing such as hot-pressing pressure, hot-pressing temperature, hot-pressing time, and ratio of PET-260 fiber and their related levels were considered, and are shown in Table 1. The experimental conditions and the results are shown in Table 2. Data obtained for TI and AP were further analyzed using Design-Expert 11 to study the relationship between experimental conditions and properties. Note that the 16th group of experimental data was abandoned in subsequent analysis because the sample lost its original network-interleaving characteristics and resembled a plastic film after hot pressing. Finally, the regression-equation prediction model of TI and AP were established to analyze response-surface values.

#### 3.3.1. Tensile Index

As shown in Table 3, analysis of variance (ANOVA) was carried out for the reduced quadratic model of TI by removing insignificant factors and influential outliers. The *p*-value and R² reveal significance and the ability to explain variance, respectively. The obtained mathematical model of TI was highly significant (*p*-value < 0.0001). Its lack-of-fit value (*p* > 0.05) indicated that the data calculated from the final model were in good agreement with the experimental data. Temperature and interaction of temperature–ratio (*p* < 0.05) were significant for TI. The adjusted R² (76.65%) represented the variance among the experimental data and the predicted R² (62.02%) represented the response of new observations. Both of them were high and their difference was <0.2, meaning that the model of TI was in reasonable proximity and more coverage in variance data. The final mathematical model is presented in Equation (6):

TI = 287.56361 − 1.32866 × A − 0.009167 × B + 0.296730 × C − 954.61603 × D + 5.375 × AD − 225.89662 × D².(6)

**Figure 9:**
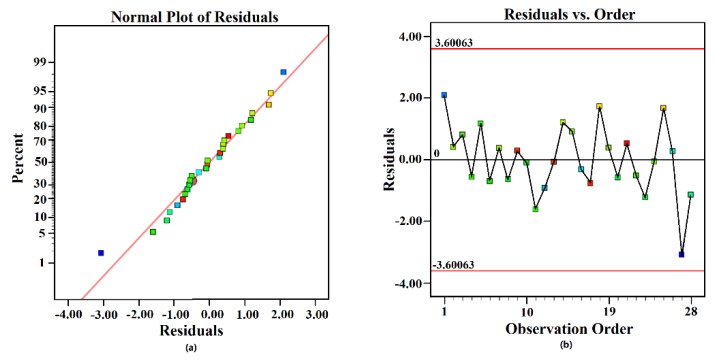
Residual plot for tensile index: (**a**) normal probability plot and (**b**) residuals vs. observation order.

Figure 9 presents the residual plot for reduced quadratic model of TI. Generally, the normal plot of residuals should be approximately a straight line and the residuals vs. observation order plot should be a random pattern. The normal probability plots of TI (Figure 9a) showed that the residuals appeared to be linearly correlated, indicating that the distribution of residuals was normal. Additionally, the residuals vs. observation order plot of TI (Figure 9b) showed that the residuals and the observation order were in random correlation, suggesting that the experimental results were not affected by observation order. Therefore, the residual plot illustrated that experimental and predicted values for the equations of the mathematical model for TI correlated well with each other.

#### 3.3.2. Air Permeability

ANOVA for reduced the quadratic model of AP is presented in Table 4. The obtained mathematical model of AP was highly significant (*p*-value < 0.0001). Its lack-of-fit value of 0.571 indicated that the model agreed well with the experimental data. Temperature and ratio were significant in determining AP (*p* < 0.01) and the interaction of time–ratio was also influential (*p* < 0.05). The adjusted R² (80.71%) and the predicted R² (73.26%) were sufficiently high, and the difference between the predicted and the adjusted R² was <0.2, meaning that the model of AP was in reasonable agreement. Thus, the final mathematical model is presented in Equation (7):

AP = 931.48276 − 2.32514 × A + 5.58419 × B − 107.7932 × C − 41.39909 × D − 13.04967 × BD + 5.80964 × C² + 797.95997 × D².(7)

**Figure 10:**
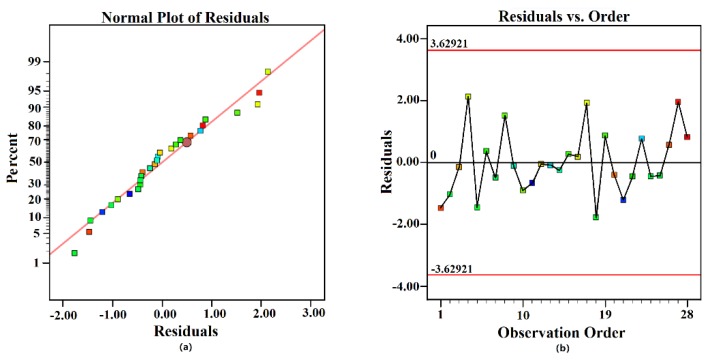
Residual plot for AP: (**a**) normal probability plot, and (**b**) residuals versus observation order.

The residual plot for reduced quadratic model of TI is presented in Figure 10. The normal plot of residuals (Figure 10a) appeared to be a linear correlation, suggesting that the residuals were normally distributed. Additionally, the residuals vs. observation order plot (Figure 10b) appeared to show a random correlation, suggesting that the observation order had no effect on the experimental result. Therefore, experimental and predicted values for the equations of mathematical model for AP correlated well with each other.

### 3.4. Performance of PNS for Separation Membrane

In this experiment, according to the regression model suggested by response surface analysis, the PNS with a minimum AP and a maximum TI were obtained by setting optimized experimental parameters (PET-260 fiber ratio, hot-pressing temperature, hot-pressing pressure, and hot-pressing time were 0.38, 220 °C, 9 MPa, and 20 s, respectively) to prove the predicted results, and the optimized PNS was used to prepare a PA composite membrane. The morphology and structure of PNS before and after hot pressing are discussed, and the properties and performance of the PA composite membrane before and after use were investigated.

#### 3.4.1. Pore Structure Analysis

PET nonwoven support is a kind of high-performance composite material with porous characteristics in its internal structure, as shown in Figure 2e and 2f. The main reasons for this were the holes and gaps between fibers formed by incompletely melted PET-260 fibers during hot pressing. Those holes and gaps directly affected the physical properties of the PET nonwoven support.

The pore-structure parameters of the PNS were measured and the results are presented in Table 5. The surface area, average pore diameter, and porosity of the nonwoven support before hot pressing were 13.34 m^2^/g, 37.89 μm, and 20.22%, respectively. After hot pressing, the three parameters of the nonwoven support decreased to 9.68 m^2^/g, 21.23 μm, and 11.28%, respectively. This finding indicates that the hot-pressed PET nonwoven support had a better interconnected structure, a smaller pore size, and a lower porosity. Fractal dimension, the product of the combination of fractal theory and material science, is also one of the most important parameters for the comprehensive evaluation of the pore-structure characteristics of composite materials. A smaller fractal dimension value means a simpler distribution of pore structure and better macroscopic properties [33]. The fractal dimension of the nonwoven support decreased from 3.02 to 2.62 after hot pressing, which further demonstrated that hot-pressing can enhance the performance of the PET nonwoven support.

#### 3.4.2. Analysis of Crystallization Characteristics

**Figure 11:**
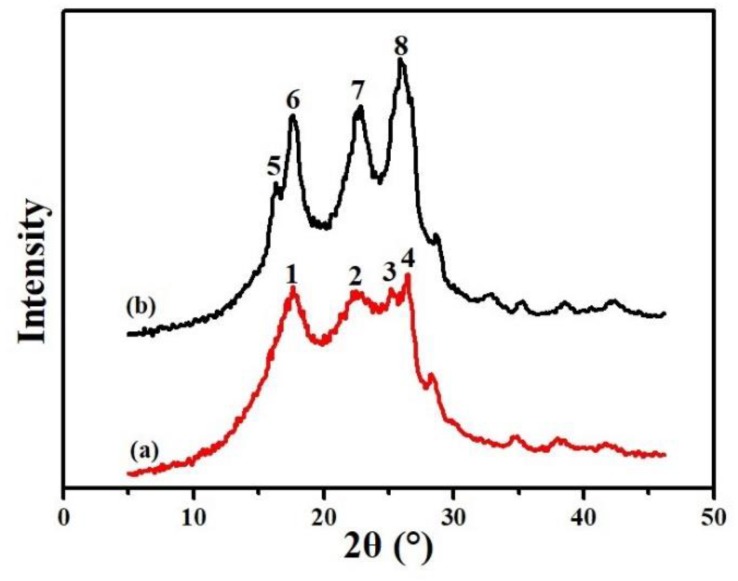
XRD patterns of the PET nonwoven support: (**a**) before hot pressing and (**b**) after hot pressing.

As a typical polymer material, the crystallinity of the PET nonwoven support had an important influence on its physical properties. In general, low crystallinity will lead to low strength of the nonwoven support, and excessive crystallization makes the whole material brittle and easy to crack under alternating external forces. The crystal structure changes of the PET nonwoven support before and after hot pressing were analyzed by an X-ray diffractometer. As shown as Figure 11, the crystallization diffraction peaks (1) changed into two narrower peaks (5,6), the broad peak (2) become a slender peak (7), and the low crystallinity diffraction peaks (3,4) developed into a crystalline diffraction peak (8) because of the gradual uniform orientation at high temperature and high pressure. Moreover, the crystallinity was 37.66% before hot pressing and 79.64% after hot pressing, which indicated that the crystalline phases of the nonwoven support were further improved during hot pressing. The main reasons for this were that temperature and pressure forced the distance between molecules of LPET-180 fibers to be decreased during hot pressing and the regular units in the low-crystallinity region tended to be evenly arranged along a certain direction. Thus, the crystallinity of LPET-180 fibers improved to enhance the mechanical strength of the PET nonwoven support.

#### 3.4.3. Performance of the PA Composite Membrane

**Figure 12:**
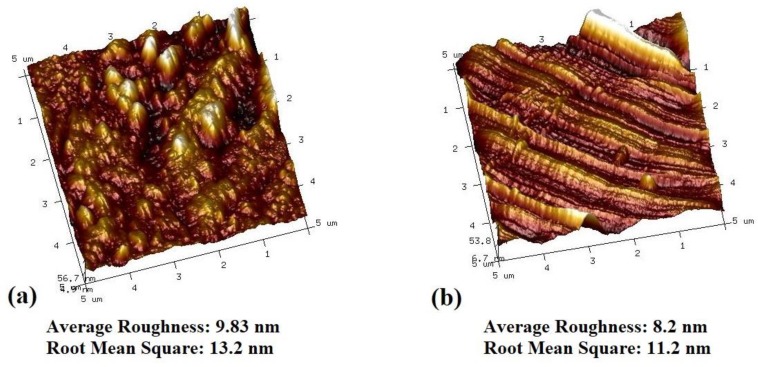
AFM of (**a**) optimized PNS and (**b**) commercial fabric support.

A comparison between the optimized PNS and a commercial fabric support was made, and the results are summarized in Table 6 and Figure 12. Compared with the commercial fabric support, the optimized PNS, even at a low weight, had smaller thickness, higher strength, and lower air permeability. Furthermore, it was found that the actual performance of the optimized PNS almost fit the prediction of the regression equation, and the differences between the actual and predicted values were 0.8 for TI and 7.05 for AP. As shown as Figure 12, the surface morphologies of the optimized PNS and the commercial fabric support showed that they had similar roughness, indicating that the homogeneity of the PNS prepared by wet-laying and hot-pressing technology was close to that of commercial fabric support.

**Figure 13:**
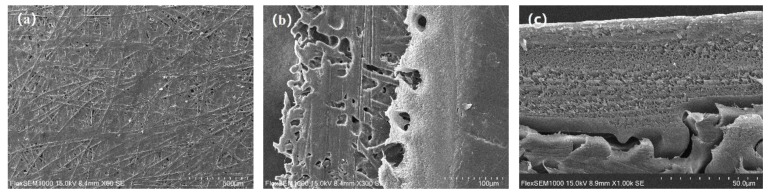
SEM images of the (**a**) back, (**b**) oblique section, and (**c**) cross-section of the polysulfone (PSF) ultrafiltration (UF) membrane.

As shown as Figure 13, solidified PSF was not found on the back of the PSF UF membrane (Figure 13a) or in the nonwoven support (Figure 13b), indicating that the PSF solution did not penetrate through the PNS, and the PSF layer was successfully formed (Figure 13c). To further characterize the practicability of the PNS, the pure water flux and retention of the PA composite membrane were measured and the results are shown in the Table 7. The flux and retention of the PA composite membrane implied that the use of the PNS for the separation membrane was feasible and practical.

**Figure 14:**
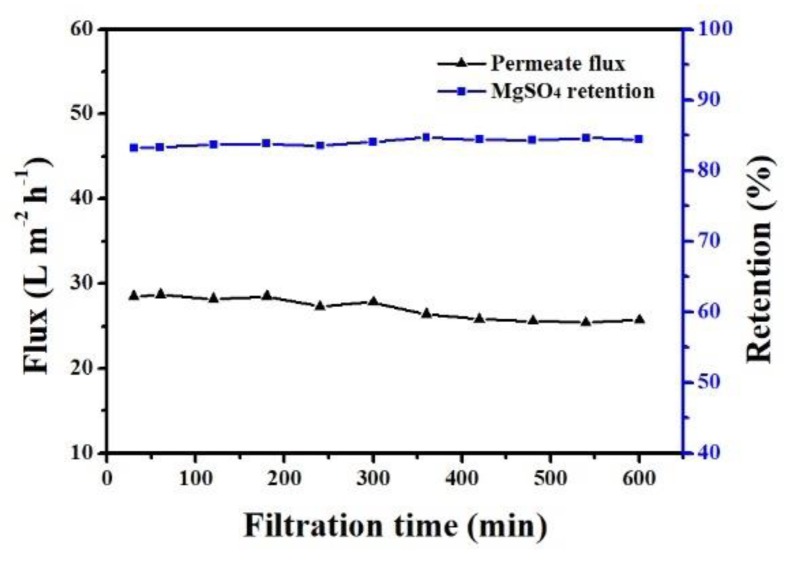
The effect of operation time on the PA composite membrane (operation conditions: 0.7 MPa, 2000 ppm MgSO_4_ solution).

**Figure 15:**
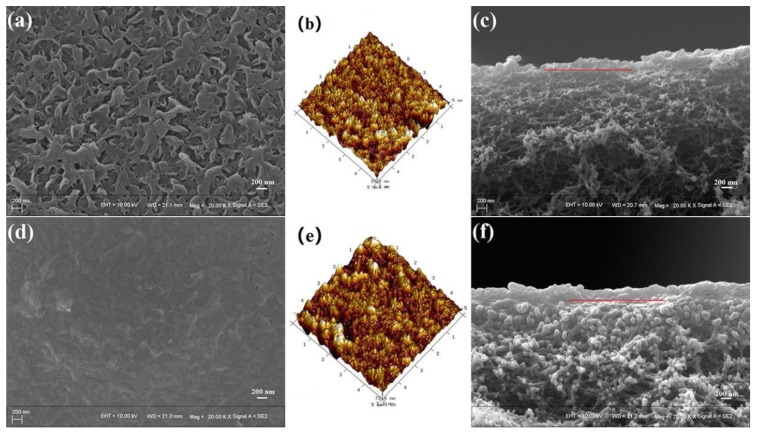
Morphologies of the PA composite membranes (**a**–**c**) before and (**d**–**f**) after use: (**a**,**d**) top surface SEM images; (**b**,**e**) top surface AFM images; (**c**,**f**) cross section SEM images (operation conditions: 0.7 MPa, 10 h).

The effects of operation time on the permeate flux and MgSO_4_ retention of the PA composite membrane were investigated, and the results are shown in Figure 14. Permeate flux slightly declined but MgSO_4_ retention increased slightly with increasing filtration time. Compared with the original membrane, the PA layer of the PA composite membranes after 10 h of use was compacted, as shown in Figure 15. As reported in many studies [34,35,36,37], this was the reason for the decrease of flux and MgSO_4_ retention with the increased filtration time.

## 4. Conclusions

The PNS which was used as a support layer for a separation membrane was successfully developed by wet-laying and hot-pressing technology. SEM, FTIR, XRD, and pore structure analyses confirmed the transformation of the PNS before and after hot pressing. The resulting PNSs were observed to have no chemical changes after hot pressing, and the fibers were bonded together by physical action. The influence of hot-pressing parameters on PNS was analyzed, and the regression-equation prediction model of TI and AP were obtained by response surface analysis. Through this approach, the PNS with a weight per square meter of 71.1 g/m^2^ at PET-260 fiber/LPET-180 fiber ratio of 0.38/0.62 (*w/w*) obtained good properties and structure after hot pressing at 220 °C under the pressure of 9 MPa for 20 s. Finally, the PNS with the best mechanical properties (TI = 52.5 N m g^−1^ and AP = 25.34 L·m^−2^·s^−1^) was obtained and showed a reinforced concrete structure with the best interconnected structure, smallest pore size, highest porosity, and greatest strength. The PNS perfectly prevented the dope solution from seeping into the support material and exhibited more advantages than spun bond PET nonwoven support. During the filtration process, the PA layer of the PA composite membranes was gradually compacted with the increased filtration time, and then permeate flux and MgSO_4_ retention gradually declined. The flux and retention of the PA composite membranes indicated that the hot-pressed wet-laid PET nonwoven support has promising prospects to be used as the support for separation membranes.

## Figures and Tables

**Table 1:** Parameter setting for the experimental design.

Code	Parameter	Levels		
		−1	0	1
A	Temperature (°C)	200	210	220
B	Time (s)	20	30	40
C	Pressure (MPa)	8	9	10
D	Ratio	0.3	0.4	0.5

**Table 2:** Experimental conditions and the results of tensile index (TI) and air permeability (AP).

Run Order	A: Temp. (°C)	B: Time (s)	C: Pressure (MPa)	D: Ratio	TI (N·m·g^−1^)	AP (L/m^2^/s)
1	200	30	9	c	32.1	105.15
2	210	20	8	0.4	45.4	62.16
3	210	30	10	0.5	44.0	95.59
4	210	30	9	0.4	42.8	84.77
5	200	30	9	0.3	42.5	64.23
6	210	30	9	0.4	42.4	69.39
7	210	30	9	0.4	45.6	60.92
8	210	30	8	0.3	40.9	74.56
9	220	30	8	0.4	53.2	50.56
10	210	40	9	0.5	41.2	78.46
11	220	30	9	0.3	42.4	24.1
12	200	30	10	0.4	34.1	91.16
13	220	40	9	0.4	52.4	45.32
14	210	30	10	0.3	46.3	53.66
15	210	40	9	0.3	45.2	71.84
16	220	30	10	0.4	54.4	26.95
17	200	20	9	0.4	35.5	86.96
18	220	30	9	0.5	53.5	86.71
19	210	40	8	0.4	48.5	63.72
20	210	20	10	0.4	45.9	72.07
21	210	20	9	0.5	40.1	100.78
22	220	20	9	0.4	54.2	28.43
23	210	40	10	0.4	43.3	68.13
24	210	20	9	0.3	39.8	41.96
25	210	30	9	0.4	44.3	61.35
26	210	30	9	0.4	49.2	61.55
27	200	30	8	0.4	36.7	103.04
28	200	40	9	0.4	29.2	108.63
29	210	30	8	0.5	38.3	110.23

**Table 3:** ANOVA for reduced quadratic model of TI (confidence level α = 0.05).

Source	Sum of Squares	df	Mean Square	*F*-Value	*p*-Value	
Model	874.94	6	145.82	15.77	<0.0001	significant
A—Temp	730.88	1	730.88	79.04	<0.0001	**
B—Time	0.1008	1	0.1008	0.0109	0.9178	
C—Pressure	0.9539	1	0.9539	0.1032	0.7512	
D—Ratio	5.20	1	5.20	0.5624	0.4616	
AD	115.56	1	115.56	12.50	0.0020	**
D²	34.80	1	34.80	3.76	0.0659	
Residual	194.18	21	9.25			
Lack of Fit	164.19	17	9.66	1.29	0.4443	not significant
Pure Error	29.99	4	7.50			
Cor Total	1069.13	27				
**Model Evaluation**						
Std. Dev.	3.04	R²		0.8184		
Mean	43.18	Adjusted R²		0.7665		
C.V. %	7.04	Predicted R²		0.6202		
		Adeq Precision		17.8744		

**Table 4:** ANOVA for reduced quadratic model of AP (confidence level α = 0.05).

Source	Sum of Squares	df	Mean Square	*F*-Value	*p*-Value	
Model	12,394.75	7	1770.68	17.13	<0.0001	significant
A—Temp	5826.02	1	5826.02	56.38	<0.0001	**
B—Time	159.28	1	159.28	1.54	0.2288	
C—Pressure	111.72	1	111.72	1.08	0.3109	
D—Ratio	5066.59	1	5066.59	49.03	<0.0001	**
BD	681.18	1	681.18	6.59	0.0184	*
C²	220.74	1	220.74	2.14	0.1594	
D²	430.04	1	430.04	4.16	0.0548	*
Residual	2066.77	20	103.34			
Lack of Fit	1648.48	16	103.03	0.9853	0.5710	not significant
Pure Error	418.29	4	104.57			
Cor Total	14,461.52	27				
**Model Evaluation**						
Std. Dev.	10.17	R²		0.8571		
Mean	72.34	Adjusted R²		0.8071		
C.V. %	14.05	Predicted R²		0.7326		
		Adeq. Precision		16.1213		

**Table 5:** Pore structure summary of the PET nonwoven support before and after hot pressing.

Pore-Structure Parameters	Total Pore Volume, (mL/g)	Surface Area, (m^2^/g)	Pore Diameter, (μm)	Porosity, (%)	Fractal Dimension
Before	0.1675 ± 0.0019	13.34 ± 0.10	37.89 ± 0.28	20.22 ± 0.34	3.02 ± 0.06
After *	0.0876 ± 0.0016	9.68 ± 0.27	21.23 ± 0.49	11.28 ± 0.45	2.62 ± 0.08

* Hot-pressing temperature, pressure, and time were 220 °C, 9 MPa, and 20 s, respectively.

**Table 6:** Performance of PET nonwoven support (PNS).

Article	Thickness (mm)	Weight (g/m^2^)	TI (N·m·g^−1^)		AP (L∙m^−2^∙s^−1^)	
			Actual	Predicted	Actual	Predicted
Optimized PNS	0.102 ± 0.008	71.1 ± 0.05	52.5 ± 2.4	51.7	25.34 ± 0.92	32.39
Commercial *	0.19	100	48		300	

* Obtained from the supplies.

**Table 7:** Filtration performance of polyamide (PA) composite membrane.

Membrane	Retention (%)		Flux (L∙m^−2^∙h^−1^)		
	MgSO_4_	NaCl	MgSO_4_	NaCl	Water
PA Composite Membrane	83.3%	41.7%	28.7	31.8	33.5
